# Establishment of an *in vitro* hairless guinea pig dermal model

**DOI:** 10.1371/journal.pone.0344659

**Published:** 2026-03-27

**Authors:** Samantha Emma Sarles, Priscilla Lee, Linnzi Wright, Daniel Angelini

**Affiliations:** United States of America Army, Combat Capabilities Command (DEVCOM) Chemical Biological Center, Aberdeen Proving Grounds, Maryland, United States of America; Anhui University of Chinese Medicine, CHINA

## Abstract

Hairless guinea pigs (HGP) make a desirable model for percutaneous exposure because they do not need to be clipped and are lower in cost and less sentient than swine. Current data leaves gaps in data translation and scaling from animal to human and from cells to whole organisms. The establishment and tracking of *in vitro* cell cultures from animals from an in-house colony allows for highly controllable and reportable data for data translation. We report the isolation, culture, methods optimization, characterization, and toxicant exposure of HGP dermal fibroblasts (HGP-DF) from the in-house HGP breeding colony at U.S. Army Combat Capabilities Development Command Chemical Biological Center (DEVCOM CBC). We successfully isolated HGP-DF from the forelimb and hindlimb skin of several HGP. Fibroblasts exited tissue within 14 days. All trypsin and media types tested for cell maintenance yielded cells with greater than 95% viability during passaging. Cells were stored under cryopreservation and exhibited acceptable viability for several passages after thawing. Fluorescently stained HGP-DF exhibited similar morphology as vendor certified human dermal fibroblasts. HGP-DF were more sensitive than human dermal fibroblasts to a toxic exposure, with median lethal concentrations of 1 mM and 0.3 mM for human cells and hairless guinea pig cells, respectively. This follows a similar trend as observed in whole organism data from the same toxicant. This work marks the first documented protocol on isolation, culture, characterization, and exposure of HGP cells. HGP-DF enable an intermediate model between human cells and *in vivo* models that may enable validation of *in vitro* models against animal models. Future work should address limitations of the present study, including evaluating phenotypic changes of cells following cryopreservation and immunohistochemistry compared to vendor certified controls to confirm dermal fibroblast phenotype.

## Introduction

Hairless guinea pigs (HGP) were first discovered at the Institut Armand Frappier (Laval, Quebec) in 1978. Some Hartley guinea pigs were born without hair except on their muzzles and feet due to a spontaneous mutation of the hairless gene. They are euthymic with functioning immune systems unlike some other hairless rodents. HGP were sent to Charles River Laboratories (Kingston, NY) in 1982 to be bred for laboratory use. They have primarily been used in dermatological studies as their skin shares several structural similarities with human skin [1]. Jung and Maibach (2014) [2] reviewed two decades of original papers that describe the permeability of chemicals through both animal and human skin and concluded that nonhuman primates, swine, and HGP are the best surrogates.

HGP have been used in U.S. Department of Defense (DoD) chemical defense research applications for more than 25 years [3]. They make a desirable model for percutaneous exposure because they do not need their hair clipped and they have a lower acquisition and housing cost than swine. A variety of studies have used HGP for percutaneous exposure of chemical warfare agents [3–5]. Since 2019, Combat Capabilities Development Command Chemical Biological Center (DEVCOM CBC) maintains the only HGP breeding colony in the U.S. or Europe.

Human skin is composed of three main layers, including the top epidermis layer, the dermis in the middle, and the underlying hypodermis. The dermal layer is the largest section in the skin, providing connective tissue which determines the skin’s mechanical properties including elasticity and tensile strength [6]. Fibroblasts are the primary cell type in the dermis and produce and regulate the extracellular matrix within the skin [7]. The cell type also plays a role in immune response, signaling of various molecules, and secretion of growth factors [7]. Fibroblasts have been well established for *in vitro* cell culture because of their rapid growth rates and ease of expansion [8]. Due to their significance in the skin and widespread use in *in vitro* modeling, fibroblasts provide utility as a model for making comparisons across species and models.

Advanced statistical models, such as Artificial Intelligence (AI) and Machine Learning (ML) models, are of interest to DoD for using historical datasets, including those from *in vivo* and human cell models, to generate human toxicology estimates. Available data leaves gaps in data translation and scaling from animal to human and from cells to whole organisms because past studies have failed to isolate inner-species phenotypic and molecular differences between species used for *in vivo* models and *in vitro* models. The availability of an intermediate model between human cells and *in vivo* animal models will enable a platform for validating *in vitro* models by characterizing them against animal models while providing the opportunity to isolate species as a factor. The establishment and tracking of *in vitro* cell cultures from DEVCOM CBC’s HGP breeding colony allows for highly controllable and reportable data for data translation. Tissue sharing from existing animal use increases the amount of data gathered from the animal. This work aims to establish an *in vitro* model of HGP dermal fibroblasts (HGP-DF) by isolating cells from pups produced by DEVCOM CBC’s HGP breeding colony for an intermediate model between animal models and human cells.

## Materials and methods

### Animal use

In conducting the research described herein, the investigators adhered to the Guide for the Care and Use of Laboratory Animals. This research was also performed in accordance with the requirements of Army Regulation 40−33, The Care and Use of Laboratory Animals in DoD Programs, and the Institutional Animal Care and Use Committee, which oversees the use of laboratory animals at DEVCOM CBC by reviewing for approval all animal use protocols. HGP pups not selected for future breeding, transferred to an approved animal use protocol for experimentation, or placed in the animal adoption program are culled immediately after weaning as part of the normal operations of the HGP breeding colony. The carcasses of four female pups were made available to investigators for this research endeavor. The pups were anesthetized with isoflurane for 1.75 min and then exposed to CO_2_ for 5 min using a HiRoad Rodent Euthanasia System. Death was confirmed by the absence of respiration and a heartbeat.

### Tissue collection

Immediately following euthanasia, pups were transferred to a hood where their skin was prepared for collection by spraying the surface of the skin with 70% ethanol and allowing it to dry. Using sterile forceps and scissors, the skin was separated from the carcass and a roughly circular sample 1 cm in diameter was cut from the underarms of each pup. Close attention was paid to remove only the skin while leaving the subcutaneous fat layer behind. Samples were collected in this fashion from the forelimbs and hindlimbs of each HGP pup. The collected samples of each animal were placed into a labeled 50 mL conical tube of sterile phosphate buffered saline (PBS) to avoid drying.

### Cell isolation

Cell isolation was performed with minor adaptions of the methods of Seluanov *et al.*, 2010 [9], which describes isolation of fibroblasts from mammalian species. After cleansing with ethanol, sample tubes were transferred to a biological safety cabinet where cell isolation was performed. A 1 cm skin sample was removed from the tube using a sterile scalpel and transferred to a 100 mm tissue culture dish. Then, using two scalpels in a scissoring motion, samples were sliced into small fragments. Seluanov *et al.*[9] recommends 1 mm fragments. As measuring pieces was impractical, skin was processed until there was a putty-like consistency, regardless of fragment size. The processed tissue was transferred to a sterile beaker with a stir bar. The 100 mm plate was washed with 10 mL of Dulbecco’s Modified Eagle Medium (DMEM)/F12 media solution with 0.14 Wunsch units/mL Liberase (Millipore Sigma, 5401119001), and 1X antibiotic/antimycotic (AA) (Millipore Sigma, A5955). The wash was then added to the beaker and sterile foil was used to cover the beaker. The covered beaker was placed on a hotplate set to 37 °C and stirred at ~200 rpm until the tissue was sufficiently digested. Digestion was determined by checking the tissue after 30 min and then every 10 min after that for the following criteria: cloudiness of the media, separation of the skin fragments from each other, and a “fuzzy” texture on the edges of the individual skin fragments. Once the tissue in the beaker was determined to be sufficiently digested, it was removed from the hotplate and suspended in digestion solution using a pipette, then transferred to a sterile 50 ml conical tube. The beaker was rinsed three times with HGP isolation media (DMEM/F12 media with 15% fetal bovine serum (FBS) (Gibco, A5256801), 1X AA). Each rinse was added to the 50 mL conical tube along with the tissue fragments. The tube was then capped, and the solution was mixed using inversion to allow the media to arrest the Liberase digestion. The solution was centrifugated at 520 g for 5 min. The supernatant was aspirated, and the resultant pellet was resuspended in 10 mL of warm HGP isolation media. Another 30 mL of warm HGP isolation media was added and the capped tube was returned to the centrifuge for another 5 min. The centrifugation process was repeated three times to ensure the removal of all traces of digestion solution. After the final round of centrifugation, the pellet was suspended in 10 mL of pre-warmed HGP isolation solution and transferred to a fresh 100 mm tissue culture plate which was covered and incubated in a tissue culture incubator at 37 °C and 5% CO_2_.

During the 2-week isolation protocol, tissue fragments remained in a tissue culture plate with HGP isolation solution. Cultures were monitored daily for fibroblast growth and color changes in the culture media. Successful isolation was indicated by a robust exodus of fibroblasts from the tissue fragments and subsequent adhesion of the fibroblasts to the plate within 2–5 days. If the plate was more than 60% confluent, media was changed and the tissue fragments were transferred to a new plate with fresh media. A lack of fibroblasts attached to the plate with a media color change indicated the presence of too many tissue fragments on the plate. This was remedied by changing the media and redistributing the tissue fragments between 2–4 additional plates with media. Any media changes were performed by centrifugation of the tissue and media at 520 g for 5 min. After which the supernatant was discarded, and the pellet was resuspended in 10 mL of fresh isolation media. If no media changes were performed by the 7-day mark, the media was changed as described above. The cells were incubated in isolation media for a total of 14 days, by which point all viable fibroblasts were assumed to have migrated from tissue fragments. After 14 days, spent media and tissue fragments were discarded through DEVCOM CBC’s biohazard waste stream.

At the conclusion of the 2-week isolation protocol, cells attached to the plate were detached with either TrypLE Express™ (Gibco™, 12604013) or Trypsin-EDTA (ATCC, PCS-999–003), centrifuged, resuspended in HGP-DF maintenance media (Eagle’s Minimum Essential Medium, ATCC, 30–2003, 15% FBS, 1X Penicillin and Streptomycin), and counted using a Vi-CELL BLU cell viability analyzer. Cells were seeded in a T75 cell culture flask at a density of 3,500 cells/cm^2^ in HGP-DF maintenance media or in fibroblast growth media FGM-2 BulletKit (Lonza, CC-3132) to support fibroblast growth and halt the proliferation of other cell types. When the cells reached ~90% confluence, they were passaged or frozen for future use. Freeze media was HGP-DF maintenance media with 10% dimethylsulfoxide (DMSO).

### Cell maintenance optimization

Type of trypsin for passaging, media type, and seeding density were optimized using a multifactorial design of experiments (DOE) matrix. Animal age was tracked but was not a controllable parameter due to the tissue sharing nature of this work.

### Trypsin type

Two types of trypsin were used during cell passaging. Either Trypsin-EDTA or TrypLE Express Enzyme (1X), no phenol red (CC-12604013), were used to detach cells from tissue culture plastic during passing. For passing cells, maintenance media was aspirated, cells were washed twice with sterile PBS, and trypsin was added. Cells were incubated at 37 °C and 5% CO_2_ for 5 min, then cells were checked under the microscope. If cells were still attached, the culture was placed back in the incubator for 2 min. This was repeated until most cells were in suspension. Once in suspension, trypsin was neutralized with cell culture media at least at a 1:1 ratio. Cells were centrifuged at 120 g for 5 min, then the supernatant was aspirated, and the pellet was resuspended in maintenance media. Cell solution was sampled, and cells were counted using a ViCell counter, which uses trypan blue as an indicator of live cells.

### Media type

Two types of media were used during cell maintenance. Either fibroblast growth media FGM-2 BulletKit or a house made HGP-DF maintenance media described above, based on the work of Seluanov *et al.*, 2010 [9], was used to maintain cells. Cells were passed when 85–90% confluent. Viability and cell density were tracked over time for cultures maintained with each media.

### Seeding density optimization and population doubling time

Four cultures were subcultured for a seeding density experiment, randomized according to the DOE matrix. Each of these cultures were seeded in 6-well plates at three seeding densities: 3,500 cells/cm^2^, 2,250 cells/ cm^2^, and 1,750 cells/ cm^2^, each in technical and biological triplicate. The four different cultures, across three different source animals and three different litters, were used at biological replicates. Data was pooled for an *N* = 12 for each seeding density. Upon plating for the experiment, all cultures were switched to house made HGP-DF maintenance media to avoid media type confounding the seeding density experiment. Cultures were checked daily under light microscopy. When ~95% confluent, cells were detached with TrypLE Express. Cells were counted and viability assessed using the ViCell cell counter. Population doubling was calculated for each sample, using Equation 1.


$$Doubling\;Rate=\frac{Duration\;[days]*\text{ln}\text{2}}{ln\frac{final\;concentation\;[\frac{cells}{mL}]}{initial\;concentration\;[\frac{cells}{mL}]}}$$ (1)

A growth curve for HGP-DF was established by seeding P4 cells at 1,750 cells/cm^2^ in a 24-well plate and monitoring cell density in technical triplicate until confluence was reached. Cells were counted on days 1, 2, 3, 6, and 8. Once cells were 100% confluent, the experiment was discontinued. For cell counting, cells were washed twice with PBS and then detached with TrypLE Express and incubated for 5 min. Cell detachment was confirmed under bright field microscopy and trypsin was neutralized with an equal volume of warm cell culture media. Cells were counted using a ViCell cell counter.

### Cryopreservation

The ability to bank HGP-DFs was assessed by freezing stocks of cells and thawing them after about 1 month in cryopreservation and assessing cell morphology and viability in the days after thawing. Stocks of cells were frozen in 1 mL aliquots at 500,000 cells/mL in HGP-DF maintenance media with 10% DMSO. The cell stocks were then placed at −80 ºC for 24–48 h, then moved to liquid nitrogen cryopreservation. To thaw, cells frozen at passage (P)4 and cells frozen directly out of the 2-week isolation protocol were removed from cryopreservation, thawed in a 37 °C water bath, and then promptly added to a T75 flask with approximately 15 mL of HGP-DF maintenance media. Media was changed 24h after thawing to remove remaining DMSO. Cultures were imaged daily for 3 days on a Zeiss Primovert bright field microscope and viability was tracked during normal cell maintenance.

### Cell expansion

HGP DF and Normal Human Dermal Fibroblasts (NHDF) (Lonza, CC-2511) were expanded for experiments comparing HDP DF to NHDF. HGP DF were cultured in FGM-2 Fibroblast Growth Medium-2 BulletKit or in house made media as described above. NHDF were cultured in FGM™-2 Fibroblast Growth Medium-2 BulletKit. Each cell type was grown in a cell culture flask and incubated at 30ºC, 5% CO_2_, 80% relative humidity until 80–90% confluent. Cells were subcultured by washing cultures two times with PBS, detaching with TrypLE Express for 3–5 minutes in physiological conditions, and neutralizing with media. Cells that were replated for continued expansion were seeded at 3500 cells/cm^2^ for NHDF and 1,750 cells/ cm^2^ for HGP DF.

### Imaging

HGP-DF P4 cultured in house made media and NHDF (P5) (Lonza, CC-2511) cultured in FGM-2 Fibroblast Growth Medium-2 BulletKit were seeded on Nunc Lab-TekChambered Coverglass (Thermo Fisher Scientific, 155383PK) at 1.4 x 10^5^ cells/mL, which corresponds to approximately 80% confluence. After 24h, culture medium was aspirated from the slides and the cells were washed three times with PBS. Then the cells were fixed by adding a 4% (w/v) solution of paraformaldehyde (PFA) in PBS and incubated for 20 min at room temperature. After which they were washed an additional three times with PBS to remove any residual fixative solution. After being fixed, the cells were permeabilized by incubating with 0.1% (v/v) Triton-X in PBS (PBS-T) and for 10 min at room temperature. Following permeabilization, the slides were again washed three times with PBS. Alexa Fluor 488 Phalloidin (Thermo Fisher, Scientific, A12379) and Hoechst nuclear stain (Thermo Scientific, 33342, ref. 62249) were added to slides and incubated at room temperature for 10 min. Slides were refrigerated for up to 1 week while protected from light before imaging.

Brightfield images were captured on either a Keyence BZ-X800 in bright field or a Zeiss Primovert bright field microscope. Scale bars were added in Keyence software. Fluorescent images were captured on a ZEISS Axio Observer confocal microscope in GFP, mRuby, and brightfield. Images were overlaid and scale and scale bars added using Zen 2.5 software.

### Exposure to Sulfur Mustard

Sulfur mustard (SM), a chemical agent with over a century of history of military use, was used to model a toxic exposure to human and HGP-DF models. All experiments were performed by qualified personnel in certified chemical fume hoods, equipped with an advanced filtration system that protects the user and the environment according to all Federal, State, and International guidelines. SM was synthesized and purified by U.S. Army DEVCOM CBC chemists in accordance with international regulations. Previously reported exposure methods [10] for SM were used for the exposure. SM was first dissolved in an organic solvent, DMSO, then the SM solution was added to FGM-2 Fibroblast Growth Medium-2 BulletKit Lonza media for a working solution of 12.54 mM SM and 0.1% DMSO. Serial dilutions were performed to achieve 6.27 mM, 3.14 mM, and 1.57 mM SM solutions. In addition to SM treatment, untreated media, a vehicle control of 0.1% DMSO in cell culture media, and a positive control of 1-part media, 1-part TRITON X-100 were exposed to cells. Cells were incubated with treatments for 24h in physiological conditions. After 24h, treatment media was removed from cells and samples were washed at least twice with PBS. All materials contaminated with chemical compound were decontaminated and disposed of through appropriate waste streams in accordance with laws and regulations.

After exposure, cell viability was assessed using alamarBlur assay. 10% alamarBlue reagent (Invitrogen, Lot 2752714) was prepared in culture media and added to each sample. Samples were incubated in physiological conditions for 2h. After incubation, solution from each well was transferred to black bottom 96-well plate. Fluorescence was read with a Spectromax plate reader at excitation wavelength 560 nm and emission wavelength 590 nm. Fluorescent signals were processed by subtracting the mean signal produced by wells with only media from the control and experimental samples to eliminate background, then normalizing data to the mean fluorescent signal of untreated control samples. The detection limit was determined by averaging the mean signal of positive control samples.

### Statistics

Descriptive statistics and graphics were generated using Microsoft Excel. Inferential statistics on growth curve data was performed using R in RStudio (version 2023.12.1). Datasets were tested for normality using a Shapiro-Wilk normality test using the Shapiro.test() function. A student’s t-test was used for hypothesis testing on normally distributed samples using the t.test() function with 0.95 significance level. A non-parametric test was performed on non-normally distributed datasets using the wilcox.test() function.

## Results

### Cell isolation

**Fig 1:**
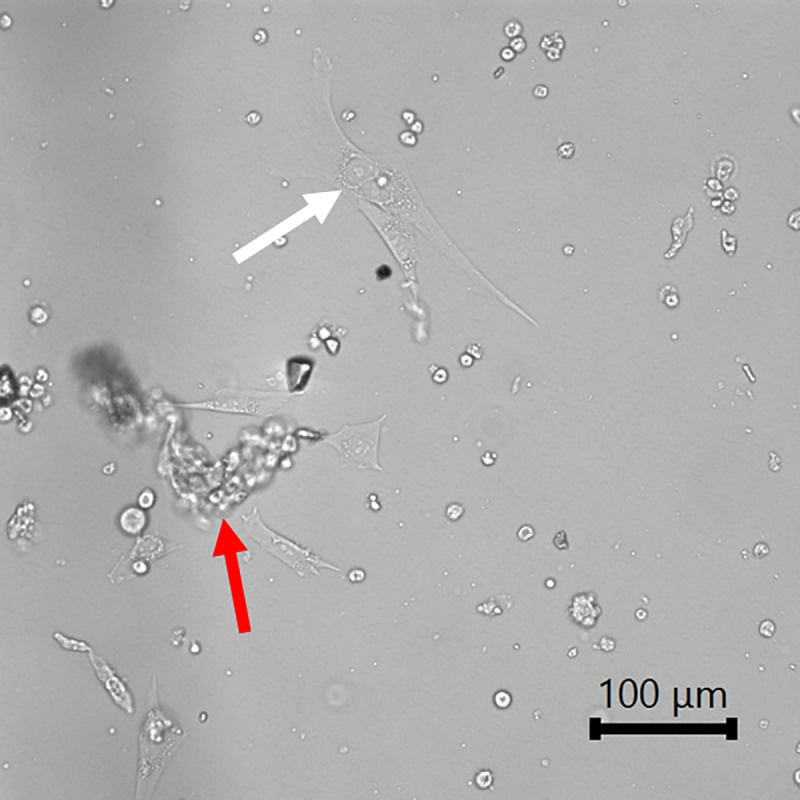
Hairless guinea pig tissue in fibroblast isolation media on Day 4. Tissue fragments with cells intact floating (red arrow). Cells with fibroblast morphology attached and spread on the petri dish (white arrow). Imaged in brightfield at 20x.

Details of animals used for cell isolation of cultures used for the data reported is given in Table 1. The dissociation start and end times of animals 0706D2 and 0723C5 were not captured. Pups were sacrificed via CO_2_ at 5–11 days old. Upon exercising the initial digestion and plating, cells and debris could be visualized floating in media under a light microscope. During the first round of isolation, tissue was collected from the forelimbs and hindlimbs of pups and plated with isolation media in four petri dishes (one per animal), media turned yellow within the first 24h in cultures. With no signs of contamination, this indicated that the amount of media in one dish could not support the large amount of living tissue in a dish. Media was changed and tissue fragments were separated into multiple dished, while tracking which animal each tissue sample came from using animal ID and “A”, “B”, “C” labels. In future isolations, tissue from a single animal was split between two or more dishes. Media was changed on Day 4, and cultures were imaged. On Day 4, tissue fragments with cells intact could be seen floating under light microscopy, shown in Fig 1 (red arrow). Additionally, on Day 4, cells with fibroblast morphology could be seen attached and spread on the bottom of the petri dish, shown in Fig 1 (white arrow).

**Table 1:** Culture metadata.

Animal ID	Sex	Age [days]	Birth Weight [g]	Weaning Weight (g)	Tissue Location	Dissociation Start Time	Dissociation End Time
0630C3	F	11	93	166	forelimbs and hindlimbs	1015	1105
0706D4	F	5	100	120	forelimbs and hindlimbs	0948	1100
0706D2	F	5	93	116	forelimbs and hindlimbs	N/A	N/A
0723C5	F	9	114	204	forelimbs and hindlimbs	N/A	N/A

**Fig 2:**
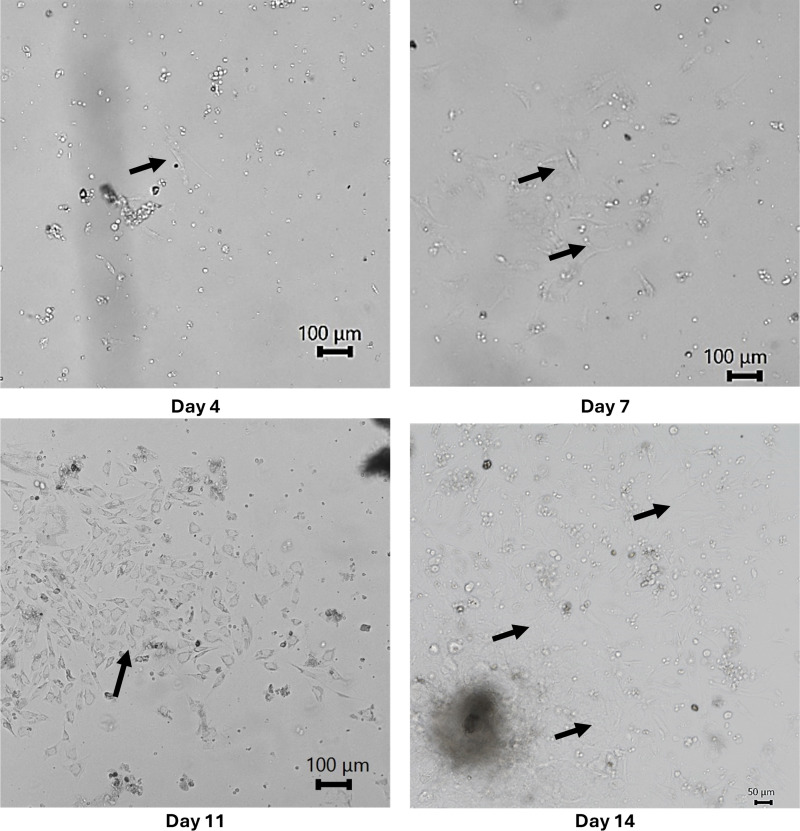
Images of culture A from animal 0630C3 on Days 4, 7, 11, and 14 of the cell isolation process. Imaged in brightfield at 20x.

Culture A from animal 0630C3 was imaged in brightfield on Days 0 (not shown), 4, 7, 11 and 14 to qualitatively monitor the rate in which cells left tissue fragments and attached to the dish. In Fig 2, long translucent fibers are live fibroblasts, several examples are pointed out with black arrows. The smaller balled up bright spots are other cell types that are dead or that did not attach to the plate. The clumps of cells are tissue that is still intact. Number of attached fibroblasts constantly increased throughout the 2 week isolation period.

### Cell maintenance optimization

Trypsin type, media type, seeding density, and animal age were tracked and analyzed using a multifactorial DOE, given in Table 2, to understand the impact of each of these factors on cell yield, viability, and proliferation. A culture received the same type of media for the duration of the study. Cultures A and D from animal 0706D2 were discarded after the first passage because there were too many cells to maintain and track given personnel and material limitations. Data from 0723C5A was not used in descriptive statistics of viability as a function of media type or trypsin type because it was frozen directly out of isolation and then thawed before regular maintenance, while other cultures were monitored without ever being frozen.

**Table 2:** Methods optimization design of experiments matrix.

Culture	Media Type	Trypsin Type P1	Trypsin Type P2	Trypsin Type P3	Trypsin Type P4+	Seeding Density Exp?	Animal Age [days]
0630C3AA	Lonza FB	TrypLE Express	Trypsin-EDTA	TrypLE Express	TrypLE Express (4), Trypsin-EDTA (5)	Y	11
0630C3AB	Lonza FB	TrypLE Express	TrypLE Express	TrypLE Express	TrypLE Express	Y	11
0706D2BC	Lonza FB	Trypsin-EDTA	TryplE Express			N	5
0706D4A	EMEM	TrypLE Express	Trypsin-EDTA	Trypsin-EDTA	TrypLE Express	Y	5
0706D2A	Lonza FB	TrypLE Express				N	5
0706D2D	EMEM	TrypLE Express				N	5
0723C5A	EMEM	TrypLE Express	TrypLE Express	TrypLE Express	TrypLE Express	Y	9

**Fig 3:**
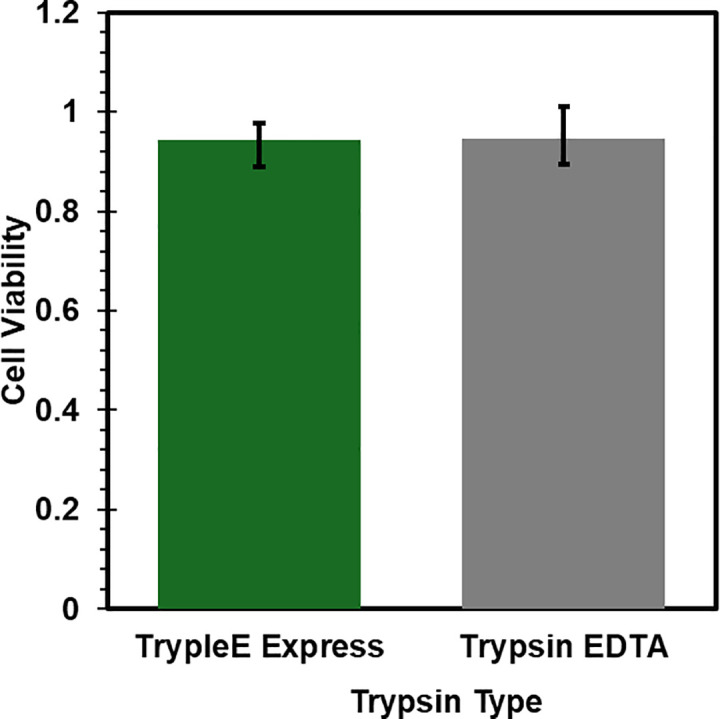
Viability of cells after being detatched with either TrypLE Express or Trypsin-EDTA. N = 12 for TrypLE Express and N = 5 for Trypsin-EDTA. Error bars are 0.95 confidence intervals.

**Fig 4:**
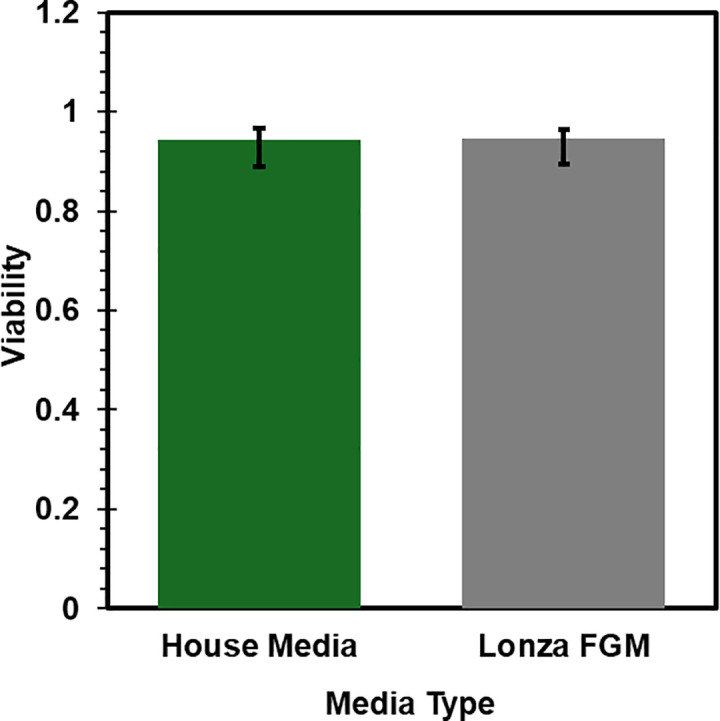
Viability of cells after being cultured with either house made media (EMEM with 15% FBS, 1X penicillin and streptomycin, and 1X sodium pyruvate) (N = 6) or FGM-2 BulletKit (Lonza, CC-3132) (N = 11). Error bars are 0.95 confidence intervals.

Fig 3 and 4 give cell viability as a function of detachment reagent and media type, respectively. Two types of trypsin, TrypLE Express and Trypsin-EDTA, were used according to manufacturer recommendations. Viability was assessed between P1 and P4 for six different cultures sourced from three different animals and is given in Fig 3. Mean cell viability after detaching cells with TrypLE Express (Fig 3) was 0.94 ± 0.05 (SD, N = 12). Mean cell viability after using Trypsin-EDTA was.94 ± 0.05 (SD, N = 5). Both types of enzymes consistently yielded cells with healthy fibroblast morphology 24h after passaging.

Two different media types, FGM-2 by Lonza and a house made fibroblast media, both described above, were used during regular cell maintenance. A culture always received the same type of media. Viability was tracked between P1 and P4 for seven different cultures sourced from four different animals and is given in Fig 4. Mean cell viability for cells cultured with house made maintenance media was 0.97 ± 0.02 (SD, N = 6). Mean cell viability in cultures that used FGM-2 was 0.93 ± 0.07 (SD, N = 11). Both types consistently yielded cells with healthy morphology and reasonable cell growth rates.

### Seeding density optimization and population doubling time

**Fig 5:**
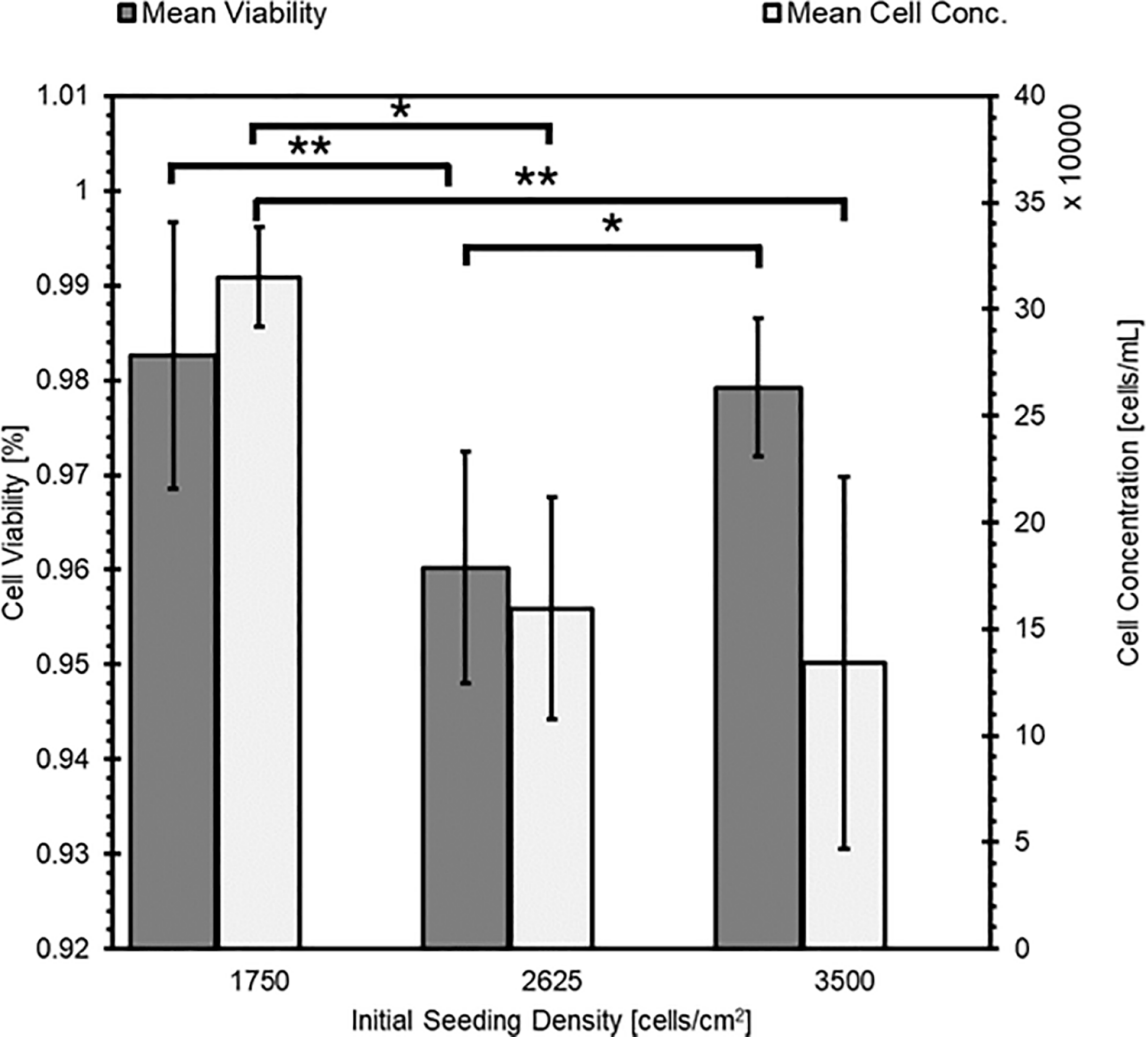
The effect of seeding density on viability and concentration of viable cells at confluence. Error bars are 0.95 confidence intervals. One asterisk (*) denotes a p-value < 0.05, two asterisks (**) denotes a p-value < 0.01.

Population doubling rate was calculated with Equation 1 using data from HGP-DF at P5, seeded at 16,800 cells/mL and cultured for 4 days. Population doubling rate is estimated to be 32h N = 9. The effect of seeding density on viability at confluence is given in Fig 5. Cells were seeded at three different densities and analyzed at ~95% confluence. In the samples seeded at 1,750 cells/mL, all three replicates from sample 0706D4A exhibited low cell density when counted despite high viability and was eliminated from analysis for both metrics. When seeded at 1,750 cells/mL, cells exhibited confluence after 7 days in culture. Mean cell viability was 0.98 ± 0.007 (SD, N = 6), and mean density of living cells was approximately 3.15 ± 1.0x10^5^. 1,750 cells/mL seeding density exhibited the highest viability and highest cell density at confluence and became confluent at about the same rate as the cultures seeded at 2,625 cells/mL. When seeded at 2,625 cells/mL, cells exhibited confluence after 7 days in culture. Mean cell viability was 0.96 ± 0.016 (SD, N = 9), and mean density of living cells was approximately 1.60 ± 0.68 x 10^5^. When seeded at 3,500 cells/mL, cells were approximately 95% confluent after four days in culture. Mean cell viability was 0.98 ± 0.019 (SD, N = 9), and mean density of living cells was approximately 1.34 ± 0.32 x 10^5^. All cell concentration and viability datasets exhibited normal distributions per a Shapiro-Wilks test. Cell viability was significantly higher in the 1,750 cell/cm^2^ condition than in the 2,625 cell/cm^2^ condition (p = 0.003) but not the 3,500 cell/cm^2^ seeding density (p = 0.632). There was a significant difference between the 2,625 cell/cm^2^ and 3,500 cell/cm^2^ seeding densities (p = 0.04). There was no significant difference in live cell concentration at confluence between the 2,625 cell/cm^2^ and 3,500 cell/cm^2^ seeding densities (p = 0.33); however, the concentration of live cells at confluence was higher in the 1,750 cell/cm^2^ than in the 2,625 cell/cm^2^ seeding density (p = 0.01) and the 3,500 cell/cm^2^ condition (p = 0.006).

### Cryopreservation

**Fig 6:**
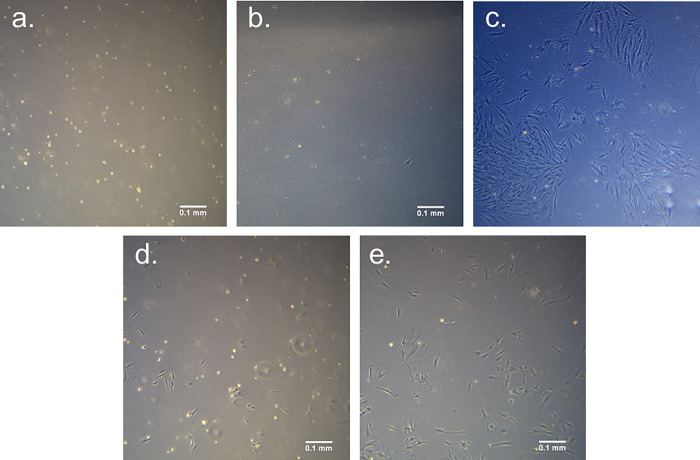
Bright field images of cultures after thawing from cryopreservation. **a.** Culture 0723C5 P0 24h after thawing. **b.** Culture 0723C5 P0 36h after thawing. **c.** Culture 0723C5 P0 48h after thawing. **d.** Culture 0706D P5 24h after thawing. **e.** Culture 0706D P5 48h after thawing.

**Fig 7:**
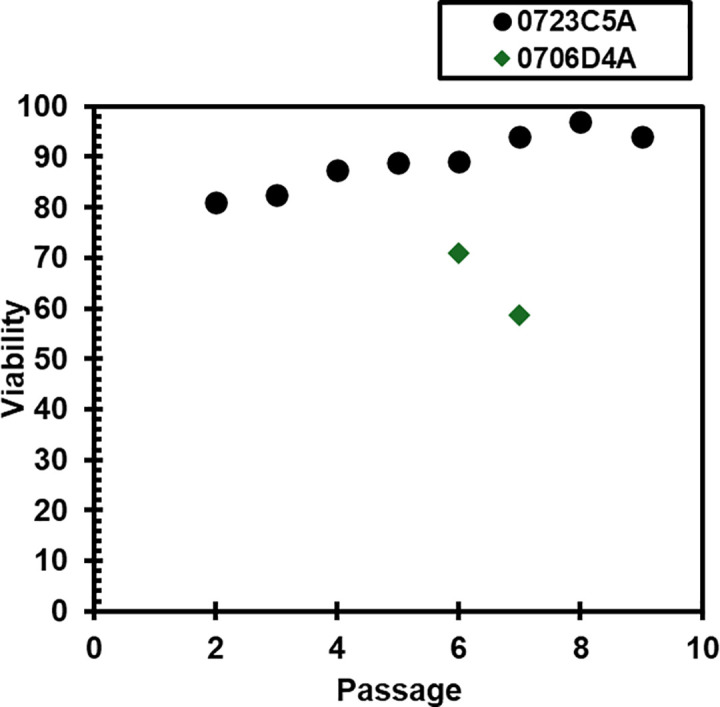
Cell viability for several passages after thawing from liquid nitrogen cryopreservation.

Two vials of cryopreserved cells, P5 culture A from animal 0706D and P0 culture A from animal 0723C5, were thawed and monitored about 1 month after freezing. Images of both cultures the first 2 days after thawing are given in Fig 6. After thawing for 24h, only a few of the P0 cells had attached to the flask, while the P5 sample had several cells attached. Within the first several days after thawing, the P0 culture had cells attached to the tissue culture plastic that did not exhibit typical fibroblast morphology. Small tissue chunks were observed within the P0 culture upon thawing, and it appeared that cell types other than fibroblasts persisted throughout the first week. After passing the cells once confluent, only cells with fibroblast morphology remained, shown in Fig 6c. Fig 7 gives cell viability for several passages after thawing cells for two cell populations. Cells frozen at P5 did not recover and no viable cells were observed after P7. In cells frozen at P1, viability did appear lower after thawing than it was in cells that were never frozen but was never lower than 80% viability with trypan blue assay.

### Proliferation

**Fig 8:**
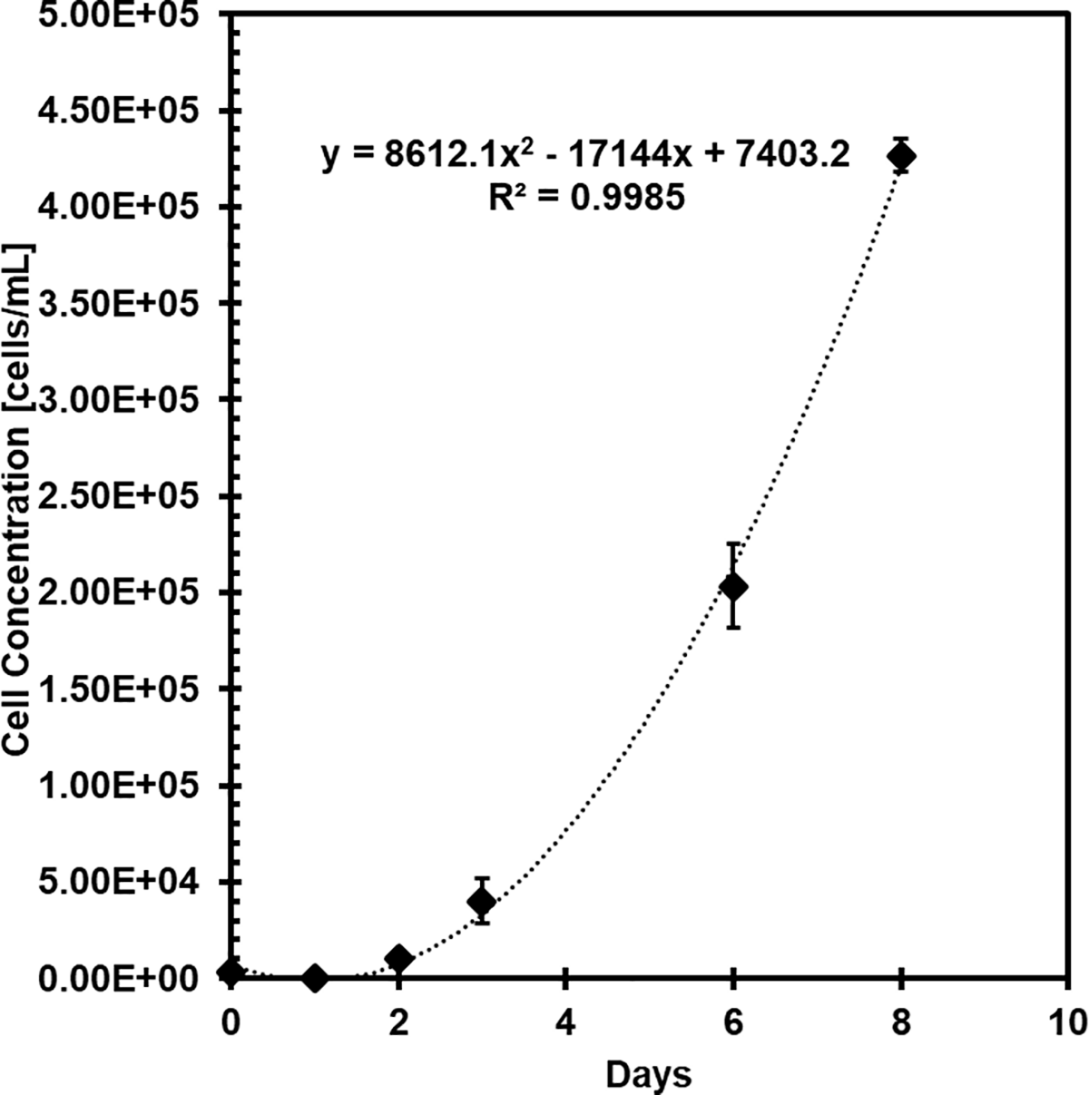
Growth curve of hairless guinea pig dermal fibroblasts seeded at P4 and analyzed at P5. N = 3 per time point, error bars are standard error.

Fig 8 gives a grown curve for HGP-DF P5 over 8 days, seeded at 3,325 cells/well in a 24-well plate with 1 mL of media. On Day 1, cells were observed under optical microscopy, but cell concentration was too low to be detected by the ViCell cell counter, hence a decrease in cell concentration between Day 0 and Day 1. From Day 2 on, cells were detected by the cell counter. Cells exhibited second order polynomial growth rate, y = 8612.1x^2^ - 17144x + 7403.2 with a coefficient of determination (R^2^) of 0.9985. Cells appeared confluent under bright field microscopy at Day 8, and the experiment was concluded. Cell cultures that were not frozen or used in experiments were maintained in flasks until spontaneous cell death occurred. 070604A failed to remain viable after P7.

### Staining

**Fig 9:**
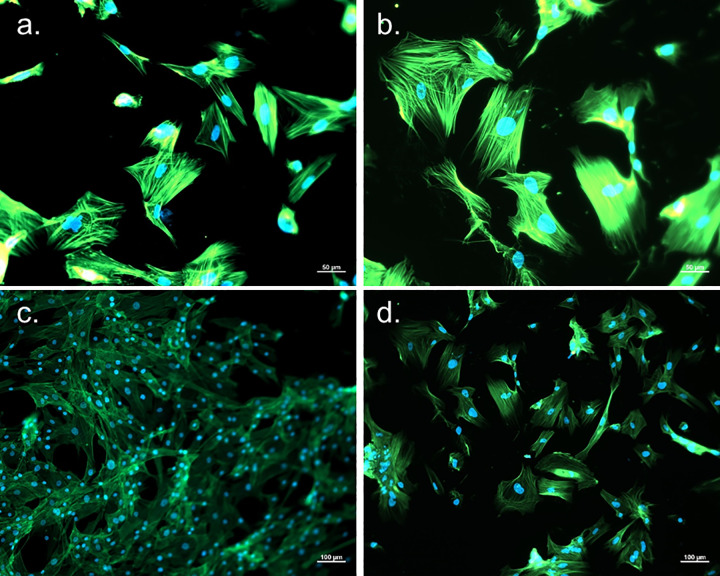
Cells stained with Cytokeratin, Multi (Epithelial Marker) Recombinant Rabbit Monoclonal Antibody (KRT/1877R, Thermo Fisher, Scientific, RBM37-1877-P0) (red), Hoechst nuclear stain (blue), and actin stained with Alexa Fluor 88 Phalloidin (Thermo Fisher, Scientific, A12379) (green), images in GFP, mRuby, and DAPI on a ZEISS Axio Observer confocal microscope using Zen 2.5 software. **a.** Normal human dermal fibroblast (Lonza, CC-2511), x20. **b.** Hairless Guinea pig dermal fibroblasts, 20x. **c.** Normal human dermal fibroblast (Lonza, CC-2511), x10. **d.** Hairless Guinea pig dermal fibroblasts, 10x.

Fig 9 gives a comparison between NHDF P5 and HGP-DF. The left column (a and c) shows NHDF, a control for what a stained fibroblast looks like. The green color is actin stained with Alexa Fluor 488 Phalloidin (Thermo Fisher, Scientific, A12379). The blue is the cell nucleus stained with Hoechst, captured in bright field.

### Exposure to a known toxicant

**Fig 10:**
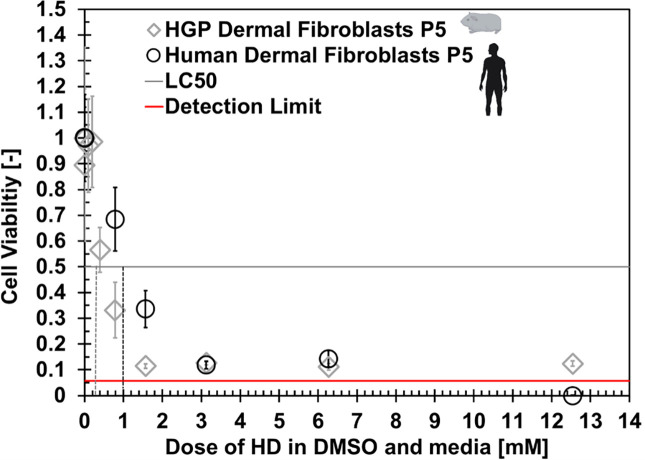
Viability of HGP and human DF exposed to sulfur mustard in 0.1% DMSO and culture media. N = 16 in experimental conditions and N = 8 for control samples. Error bars are 0.95 confidence intervals. Icons from NIH BioArt.

Fig 10 shows dose response curves for HGP-DF and NHDF exposed to SM in DMSO and culture media. The median lethal concentration (LC_50_) of the two models are approximately 0.3 mM and 1 mM SM for the HGP-DF model and the NHDF model, respectively. In preliminary experiments, it was observed that the HGP-DF viability approached the detection limit of the alamarBlue assay at lower concentrations than the NHDF, so additional serial dilutions of the SM in DMSO were performed in culture media to achieve lower concentrations and elicit a dose-response curve for the HGP-DF model. Each sample was normalized to an untreated control of its matched species. A Shapiro-wilk test was performed on each of the datasets to test normality prior to hypothesis testing. In the HGP-DF model, the vehicle control and the 0.783 mM SM concentration exhibited non-normality. In the NHDF model, the vehicle control and the 1.57 mM SM concentration data exhibited non-normality. A Wilcoxon signed-rank test was used for hypothesis testing of non-normal datasets while a student’s t-test was used for normally distributed data.

The viability of the vehicle control (0.1% DMSO in culture media) was 89 ± 35% (SD) in HGP-DF and 100 ± 14% (SD) in the NHDF model. The difference was not statistically significant. HGP-DF were exposed to three lower concentrations of SM than the NHDF due to low viability. HGP-DF exhibited viability of 0.97 ± 0.34 (SD) at 0.99 mM SM, 0.99 ± 0.33 (SD) at 0.198 mM SM, and 0.56 ± 0.16 (SD) at 0.395 mM SM. At the 0.783 mM SM concentration, viability of HGP-DF was 0.33 ± 0.20 (SD) while viability was 0.68 ± 0.22 (SD) at the same concentration. Results were statistically significant (p < 0.0001). Viability at the 1.57 mM concentration was higher in the NHDF model, with 0.34 ± 0.13 (SD) viability, than the HGP-DF model with 0.11 ± 0.21 (SD) viability; results were significant (p < 0.001). There was not statistical significance between models at 3.13 mM SM and 6.27 mM SM, with viability falling between 0.10–0.15 for both models at both concentrations, approaching the detection limit of the assay. The mean viability of NHDF at the 12.54 mM concentration was below the detection limit. Sample sizes were as follows: N = 16 in experimental conditions and N = 8 for control samples.

## Discussion

The described work marks the first published protocol on isolation, culture, and characterization of HGP cells, an animal model used in chemical warfare agent (CWA) research by the DoD. We also conduct the first controlled comparison between human and HGP cells to a toxic exposure. The establishment and characterization of *in vitro* culture of HGP-DF, a cell type abundant in the skin, provides a new capability for researchers that may produce datasets to aid in translation and scaling of *in vitro* and *in vivo* data, especially with the emergence of advanced statistical and computational models, including AI and ML. Further, the protocol described provides an update to a previously published protocol for “Establishing Primary Adult Fibroblast Cultures From Rodents” that appeared in literature in 2010 [9].

Isolation and culture of cells was completed on pups 5−11 days old with tissue collected from the forelimbs and hindlimbs of each animal. During the isolation period, if media began to turn yellow, cultures were checked for contamination or overcrowding. Though more common in wild caught specimens than in laboratory animals, the presence of any fungi or worms on the plate should result in the plate being discarded. This did not occur during any of the isolations performed. Following the isolation period which used a broad spectrum media, fibroblasts remained viable when cultured with both a commercial, proprietary culture media, FGM-2 by Lonza, and with a house made EMEM based maintenance media. Similarly, cells exhibited good viability and appeared to be in good health based on visual observation after passage with both TrypLE Express and Trypsin-EDTA. Based on this finding, researchers proceeded with regular maintenance of the house made media and TrypLE express because of the lower cost and widespread availability of the reagents.

Population doubling rate was 32h at P2. This is well aligned with prior work that isolated fetal guinea pig dermal fibroblasts and reported population doubling rate of 23h at P2 and 30h at P8 [11]. Based on the data collected after seeding cells at variable densities, the recommended seeding density of HGP-DF is 1,750 cells/cm^2^ for highest viability and live cell concentration after 7 days; however, all three seeding densities tested yielded viability greater than 95% at confluence and higher seeding density is acceptable to reach confluence faster as needed. Based on qualitative observations made during this work, our recommendation is to not culture cells beyond six population doublings.

HGP-DF were stored under cryopreservation and then thawed for further expansion and use. Small tissue chunks were observed upon thawing in cultures that were frozen directly out of the isolation protocol and not first moved to fibroblast maintenance media. Additionally, it appeared that cell types other than fibroblasts persisted within the first week after thawing. After passing cells, only cells with fibroblast morphology remained. Based on these observations, it is recommended to culture cells in maintenance media for at least 7 days before freezing stocks of cells. Cell viability was generally lower after thawing from cryopreservation than it was in cells that were never frozen (Fig 3,4). Despite this, viability remained above 80% when frozen at a low passage, an acceptable value by most standards [12]. Still, without quantifying possible changes in phenotype following freezing and thawing, it is recommended to use cells that have not been frozen for experiments. Future work should address this limitation by evaluating phenotypic differences between cells before and after cryopreservation.

Fluorescent staining with Alexa Fluor 488 Phalloidin and Hoechst nuclear stain allowed visual observation of cell morphology. Staining showed that the isolated HGP-DF exhibited morphology nearly identical to vendor certified NHDF. This finding suggests successful isolation of HGP-DF that maintained a fibroblast phenotype in culture. HGP-DF appeared larger than NHDF in fluorescent images. Aside from interspecies differences, NHDF were cultured in Lonza FGM while the stained HGP-DF cultures imaged were cultured in house made media. Possibly, the difference in media impacted phenotype of the cells and may warrant further investigation. Future work will include immunohistochemistry using antibody stains against vendor certified controls to further confirm dermal fibroblast phenotype.

Animal age was treated as a lurking variable and tracked, but not controlled or analyzed, as animal age at euthanasia was not a factor that researchers could control because of the tissue sharing nature of this work. Future work may examine the impact of animal age at cull on cell yield after isolation.

Exposure to SM, a known toxicant in both humans and HGP [4], produced the first comparison between human and HGP *in vitro* models under controlled conditions. SM is a vesicant agent with primary hazard to the lungs, skin, and eyes. The mechanism of action of SM is somewhat known, with DNA damage disrupting protease activity leading to downstream inflammation and cell death, but specific pathways and biological targets have not been concretely identified [13]. *In vitro* models from both species responded to SM similarly at higher concentrations of SM exposure, HGP-DF were more sensitive to than NHDF at lower concentrations with an LC_50_ around 0.3 mM while the LC_50_ in the NHDF model was around 1 mM. The LC_50_ HGP model agrees more closely with prior work that exposed NHDF to SM diluted in ethanol and media for 24h and produced an LC_50_ of 0.162 mM [14]. The trend we observed of HGP-DF being more sensitive to SM than NHDF under the same exposure conditions qualitatively mimics documented whole organism response. While there is no published controlled experiment that allows direct comparison between guinea pig and human response to SM, inhalation LCt_50_ for each species is available in the literature. Langenberg established an inhalation LCt_50_ for HGP to be 800 mg min/m^3^. Meanwhile, the most up to date estimate for human inhalation median lethal concentration is 1,000 mg min/m^3^ [15]. This finding is encouraging when considering the potential of *in vitro* animal models to aid in translation between *in vitro* and *in vivo* models, especially with the availability of advanced statistical frameworks that utilize AI/ML.

This study establishes an isolation protocol for hairless guinea pig dermal fibroblasts and characterization of cell growth under a variety of culture conditions. A limitation of this work is that phenotype was not verified through quantitative methods. This should be addressed in future work with single cell RNA sequencing or immunohistochemistry. Other limitations of this work include lack of analysis of phenotype following cryopreservation and the effect of animal age was not tested.

The reported work describes successful isolation, culture, methods optimization, characterization, and toxicant exposure of HGP-DF from an in-house breeding colony at DEVCOM CBC. This capability enables an intermediate model between human cells and animal models that may enable comparison of *in vitro* models against animal models and provides methods to experimentally isolate interspecies variation. In the future, collaborations between domain experts and data scientists should inform practical utility of this model through careful design of experiments that will best fill gaps in training data for AI. This capability will increase the utility of input data for advanced models, such as AI and ML, and will advance the mission of the DoD CWA defense program by expanding the use of historical datasets, including those from *in vivo* and human cell models, to generate human toxicology estimates.
